# Molecular evidence of haemosporidian infections in tawny owls (*Strix aluco)* from central Norway

**DOI:** 10.1016/j.ijppaw.2026.101208

**Published:** 2026-02-09

**Authors:** Andrea S. Ingebretsen, Siren C. Svanes, Ingvild B. Kroglund, Oddmund Kleven, Rita Santos, Jan Eivind Østnes, Helena Costa

**Affiliations:** a Faculty of Biosciences and Aquaculture, Nord University, Universitetsalléen 11, Bodø, NO–8026, Norway; b Faculty of Biosciences and Aquaculture, Nord University, Skolegata 22, Steinkjer, NO–7713, Norway; c Norwegian Institute for Nature Research (NINA), Høgskoleringen 9, NO-7034, Trondheim, Norway; d CIBIO, Research Centre in Biodiversity and Genetic Resources, InBIO Associated Lab, Vairão Campus, University of Porto, Vairão, 4485-661, Portugal; e Department of Biology, Faculty of Sciences, University of Porto, Porto, 4099-002, Portugal; f BIOPOLIS Program in Genomics, Biodiversity and Land Planning, CIBIO, Vairão Campus, Vairão, 4485-661, Portugal

**Keywords:** *Leucocytozoon*, *Haemoproteus*, *Plasmodium*, Avian malaria, Wildlife health, Vector-borne disease

## Abstract

Haemosporidians, including *Leucocytozoon* spp., *Plasmodium* spp., and *Haemoproteus* spp., are vector-borne parasites that cause avian malaria and malaria-like diseases in birds. These infections can result in anaemia, reduced body condition, impaired reproductive success, and mortality, thereby acting as selective agents within host populations. Climate change is expected to influence dynamics of some haemosporidians, particularly in northern regions, by altering vector distributions and extending transmission seasons. In this study, tawny owls (*Strix aluco*; n = 27) from central Norway, were screened for *Leucocytozoon* spp., *Haemoproteus* spp*.,*and *Plasmodium* spp., using polymerase chain reaction (PCR). Samples were also screened for herpesvirus and *Chlamydia* spp., via PCR, to assess potential co-infections. Ten individuals (37%) tested positive for *Leucocytozoon* spp., with sequencing confirming the lineages STAL3 and STAL1, both previously detected in raptors. While seven individuals (26%) were positive in the *Haemoproteus/Plasmodium* PCR, sequencing was unsuccessful and infection could not be confirmed. Neither herpesvirus nor *Chlamydia* spp. were detected. This study provides the first molecular evidence of haemosporidian infections in raptors in Norway, underscoring the importance of continued surveillance of avian haemosporidians in resident northern species, to detect potential climate-driven changes in infection prevalence and associated health impacts.

## Highlights

• First molecular evidence of haemosporidians in Norwegian raptors.
• *Leucocytozoon* detected in 37% and identified as the lineages STAL3 and STAL1.
• 26% PCR-positive for *Haemoproteus/Plasmodium* spp., but failed sequencing.
• No evidence of herpesvirus or *Chlamydia* sp. infections.
• Supports ongoing surveillance of avian blood parasites in northern regions.

## Graphical abstract

**Figure:**
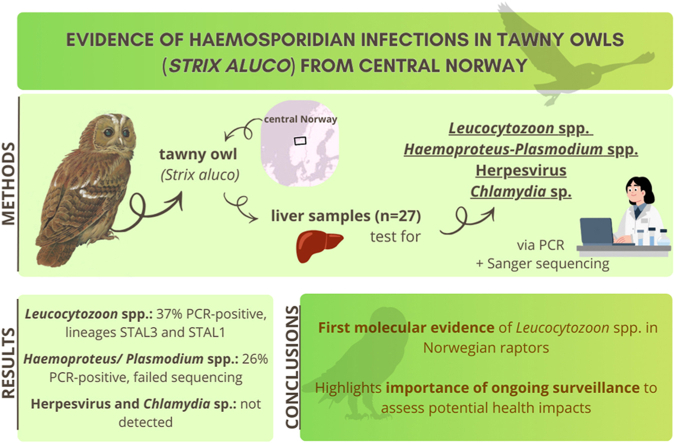

## 1 Introduction

Haemosporidian protozoa, including *Plasmodium*, *Haemoproteus*, and *Leucocytozoon* are among the most widespread avian pathogens (Valkiūnas, 2004; Foster and Walker, 2019). These parasites are transmitted by dipteran vectors such as mosquitoes (*Culicidae*), biting midges (*Ceratopogonidae*), and black flies (*Simuliidae*), and are responsible for a range of avian vector-borne diseases, including avian malaria and malaria-like disease (Wilkinson et al., 2016; Valkiūnas and Iezhova, 2018; Bowman, 2021). Haemosporidian infections can cause anaemia, reduced body condition, impaired reproductive success, and even mortality (Korpimäki et al., 1993; Atkinson and Samuel, 2010). In wild bird populations, chronic infections may also affect migration ability, long-term fitness, and susceptibility to secondary infections (Marzal et al., 2005; Asghar et al., 2011). Severe infections have been linked to local population declines among raptors and passerines (Krone et al., 2008a, Krone et al., 2008b; Rogers, 2012).

The distribution and prevalence of haemosporidian parasites are largely influenced by climate, vector availability, and host ecology. In temperate and subarctic regions, parasite transmission has traditionally been limited by short vector seasons and low temperatures that constrain sporogony in the insect host (Wilkinson et al., 2016). However, recent evidence indicates that some haemosporidians are expanding northward as global temperatures rise (Garamszegi, 2011; Loiseau et al., 2012). This latitudinal shift is supported by reports of increasing parasite prevalence in Scandinavian bird populations, suggesting that vector-borne diseases may establish more stable transmission cycles in previously unsuitable northern habitats (Hellgren et al., 2008; Sehgal, 2015; Kleinschmidt et al., 2022). Other environmental and demographic factors such as humidity, season, host age, sex and contact with other species can potentially also be key determinants of infection dynamics (Martínez-de la Puente et al., 2011; LaPointe et al., 2012; van Rooyen et al., 2013; Donaldson et al., 2024). Understanding how these factors interact in high-latitude ecosystems is crucial for predicting vector presence and parasite transmission under ongoing climate changes.

In addition to haemosporidians, other avian pathogens such as *Chlamydia* and herpesvirus are of increasing concern due to their potential to cause co-infections and cross-species transmission. *Chlamydia psittaci* is the primary agent of avian chlamydiosis and a well-known zoonotic pathogen, documented in over 450 bird species from 30 orders worldwide (Kaleta, 2021; Ravichandran et al., 2021; Van Wettere, 2023). The bacterium can cause respiratory disease and reproductive disorders in birds and may pose health risks to humans who handle infected individuals (Sachse et al., 2015; Ravichandran et al., 2021; Van Wettere, 2023). Wild birds are also known reservoirs of herpesviruses, which have been associated with several avian diseases including Marek's disease, Duck virus enteritis, avian infectious laryngotracheitis, and Pacheco's disease (Tomaszewski et al., 2001; Amery-Gale et al., 2018; WOAH, 2018; Gowthaman et al., 2020; WOAH, 2021). Although such infections are less frequently documented in wild raptors, they may contribute to immunosuppression and increase vulnerability to other pathogens such as haemosporidians (Atkinson and van Riper, 1991).

Owls (order Strigiformes) play a crucial ecological role as nocturnal predators by helping regulate prey populations and maintain ecological balance within ecosystems (Donázar et al., 2016). As top avian predators, they are considered important bioindicators of environmental health, particularly in forested and agricultural landscapes. Despite their ecological significance, little is known about the prevalence and effects of infectious diseases in owls in Norway, and few studies have assessed how pathogens may affect their survival or population dynamics.

The tawny owl (*Strix aluco*) is a common and largely sedentary owl species distributed across Europe and into southern and central Norway (Holt et al., 2025). As a generalist predator that uses both rural and urban habitats (Holt et al., 2025), it is frequently exposed to diverse prey species and potential pathogen reservoirs. Due to its limited territories, short natal dispersal distances and limited movements, the tawny owl is a valuable model for studying the local persistence and transmission of vector-borne parasites. Although haemoparasites have been recorded in several European owl species, including tawny owls (Krone et al., 2008a, Krone et al., 2008b; Santos et al., 2025), data from Norwegian populations remain scarce. In particular, there are no published molecular records of haemosporidian infections or other pathogens in owls from Norway. Establishing such baseline information will be essential for future surveillance programs and for assessing the potential effects of emerging pathogens in changing climates.

The present study aimed to screen tawny owls from central Norway for the presence of *Plasmodium*, *Haemoproteus*, *Leucocytozoon*, herpesvirus, and *Chlamydia* spp. infections, and to discuss the ecological and conservation implications of haemosporidian presence in high-latitude environments.

## 2 Materials and methods

### 2.1 Study population, sampling, and sample characterisation

**Fig. 1:**
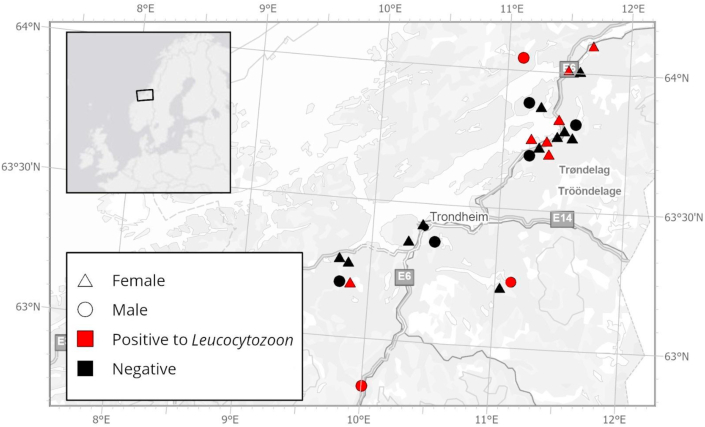
Map illustrating the geographic position in central Norway where cadavers of tawny owls (*Strix aluco*) were collected, sex of the individuals and results of the pathogen screening.

Between 2009 and 2022, liver samples were collected from 27 dead tawny owls found opportunistically in Trøndelag, central Norway, their northernmost breeding range in Europe (Fig. 1). These included 19 females and eight males, of which four individuals were in their first calendar year, two in their second calendar year, 20 were adults, and one was unknown. The cause of death was verified for eight of them, and included road traffic accident for three owls, predation for two, electrocution for one, and two were found in traps. Further details on sex, age class, cause of death and origin of the individuals can be consulted in Supplementary Table 1.

After collection, samples were stored at −20 °C, transported to the laboratory at Nord University, Bodø (Norway), and stored at −80 °C until analysis.

### 2.2 DNA extraction and sex determination

DNA was extracted from approximately 100 mg of homogenised liver tissue using the DNeasy Blood & Tissue Kit (QIAGEN, Hilden, Germany), according to the manufacturer's instructions. DNA purity and concentration were measured with NanoDrop One (Thermo Fisher Scientific).

Sex determination was performed via PCR at Norwegian Institute for Nature Research (NINA), Trondheim, Norway, using the marker Z37B (Dawson et al., 2015) that amplifies short fragments on both the W and Z chromosomes (female birds – ZW, male birds ZZ). The PCR reaction was performed with Qiagen's Multiplex PCR Kit, following the manufacturer's protocol, but using 8.4 μL final volume. The forward primer was fluoro-labelled (PET). PCR products were mixed with GeneScan 500 LIZ (Applied Biosystems) size standard and Hi-Di formamide. Alleles were separated using capillary electrophoresis on an ABI 3500*xl* Genetic Analyzer and sizes sizes (W = 97 base-pairs, Z = 99 base-pairs) assigned using GeneMapper v6.0 software (Applied Biosystems).

### 2.3 PCR amplification

#### 2.3.1 Haemosporidians

A nested PCR was performed to detect haemosporidian parasites by targeting the mitochondrial *cytb* gene, according to Bensch et al. (2000) and Hellgren et al. (2004). The first reaction amplified *Haemoproteus*, *Plasmodium*, and *Leucocytozoon* DNA, followed by a nested reaction with different sets of primers to amplify either *Haemoproteus-Plasmodium* or *Leucocytozoon DNA.* The first reaction was carried out in a final volume of 25 μL, including 12.5 μL of AmpliTaq Gold 360 Master Mix (Applied Biosystems); 2 μL of primer HaemNFI; 2 μL of primer HaemNR3; and 7.5 μL of RNAse-free water, to which 1 μL of DNA was added. A first nested reaction, conducted to detect *Haemoproteus-Plasmodium* DNA*,* was accomplished in a final volume of 25 μL, with 12.5 μL of AmpliTaq Gold 360 Master Mix (Applied Biosystems); 2 μL of primer HaemF; 2 μL of primer HaemR2; and 6.5 μL of RNAse-free water, to which 2 μL of the product of the first reaction was added (Supplementary Table 1). A second nested reaction, conducted to detect *Leucocytozoon* DNA, was carried out in a final volume of 25 μL, including 12.5 μL of AmpliTaq Gold 360 Master Mix (Applied Biosystems); 2 μL of primer HaemFL; 2 μL of primer HaemR2L; and 6.5 μL of RNAse-free water, to which 2 μL of the product of the first reaction was added (Supplementary Table 1). A positive control, containing DNA obtained from a barn owl (*Tyto alba*) with a verified mixed infection with *Haemoproteus/Plasmodium* spp. and *Leucocytozoon* spp. (Santos et al., 2025), and a negative control (RNAse-free water) were added to each reaction. The thermocycle conditions (BIO-RAD C1000 Touch Thermo Cycler, Hercules, California, USA) used for all reactions included an initial denaturation step at 95 °C for 5 min, followed by 35 cycles of 95 °C for 30 s, 50 °C for 30 s, and 72 °C for 45 s, with a final extension step at 72 °C for 7 min. PCR products were analysed on a 1.2% agarose gel and visualised using BIO-RAD ChemiDoc MP Imaging System (Hercules, California, USA).

#### 2.3.2 *Chlamydia* spp.

A PCR was performed to detect *Chlamydia* spp. DNA by targeting the 16S rRNA gene, according to Robertson et al. (2009). The reaction was carried out in a final volume of 25 μL, including 12.5 μL of AmpliTaq Gold 360 Master Mix (Applied Biosystems); 2 μL of primer 16SG-F; 2 μL of primer 16SG-R; and 7.5 μL of RNAse-free water, to which 1 μL of DNA was added (Supplementary Table 1). A positive control, containing *Chlamydia* DNA from a human sample, and a negative control (RNAse-free water) were added to the reaction. The thermocycle conditions (BIO-RAD C1000 Touch Thermo Cycler, Hercules, California, USA) included an initial denaturation step at 95 °C for 5 min, followed by 35 cycles of 95 °C for 30 s, 58 °C for 30 s, and 72 °C for 30 s, with a final extension step at 72 °C for 5 min. PCR products were analysed on a 1.2% agarose gel and visualised using BIO-RAD ChemiDoc MP Imaging System (Hercules, California, USA).

#### 2.3.3 Herpesvirus

A nested PCR was performed to detect herpesvirus DNA by targeting three conserved motifs in a region of the viral DNA polymerase gene, according to VanDevanter et al. (1996). The first reaction was carried out in a final volume of 25 μL, including 12 μL of AmpliTaq Gold 360 Master Mix (Applied Biosystems); 1 μL of primer DFA; 1 μL of primer ILK; 1 μl of primer KG1; and 7 μL of RNAse-free water, to which 3 μL of DNA was added (Supplementary Table 1). The second reaction was carried out in a final volume of 25 μL, including 12.5 μL of AmpliTaq Gold 360 Master Mix (Applied Biosystems); 1 μL of primer TGV; 1 μL of primer IYG; and 8 μL of RNAse-free water, to which 2.5 μL of product from the first reaction was added (Supplementary Table 1). A positive control, containing DNA obtained from a bat herpesvirus (Costa et al., unpublished), and a negative control (RNAse-free water) were added to each reaction. The thermocycle conditions (BIO-RAD C1000 Touch Thermo Cycler, Hercules, California, USA) used for both reactions included an initial denaturation step at 94 °C for 5 min, followed by 45 cycles of 94 °C for 30 s, 46 °C for 1 min, and 72 °C for 1 min, with a final extension step at 72 °C for 7 min. PCR products were analysed on a 1.2% agarose gel and visualised using BIO-RAD ChemiDoc MP Imaging System (Hercules, California, USA).

### 2.4 Sequencing

All amplicons of expected size were purified and prepared for Sanger sequencing using the QIAquick Gel Extraction (Qiagen) and the BigDye Terminator v3.1 (Thermo Fisher Scientific) kits, according to the manufacturers’ instructions. Samples were sent for sequencing at the University Hospital of North Norway, in Tromsø (Universitetssykehuset Nord-Norge HF).

The forward and reverse sequences were aligned by the MUSCLE method from MEGA 11 software, and the consensus sequences were obtained. The consensus nucleotide sequences obtained were subjected to a BLAST (Basic Local Alignment Search Tool) search (http://blast.ncbi.nlm.nih.gov/Blast.cgi) for comparison with other sequences available at the database GenBank NCBI.

### 2.5 Statistical analysis

Associations between infection status (presence/absence) and sex, age class, year of collection, and sampling location were assessed using Fisher's exact tests due to small sample size. All tests were two-sided, and statistical significance was set at p < 0.05. All statistical analyses were conducted in RStudio (R version 2025.09.2 + 418).

## 3 Results

### 3.1 Leucocytozoon spp.

*Leucocytozoon* spp. DNA was detected via PCR in ten of 27 samples (37%) (Fig. 1; Supplementary Table 2). BLAST searches and submission to the Malavi database indicated that seven of the positive *Leucocytozoon* samples were identical (100% similarity) to the lineage STAL3, previously reported in Strigiform hosts in Europe, including tawny owls in Austria (MK652258.1; ON932283.1) and a Eurasian pygmy owl (*Glaucidium passerinum*) in Lithuania (ON932273.1). Three of the positive samples were identical (100% similarity) to the *Leucocytozoon* lineage STAL1 detected in tawny owls from Austria (ON932243.1) and Germany (EF607285.1). Overall, the *Leucocytozoon* sequences obtained in this study were more closely related to lineages found in other Strigiformes hosts across central and eastern Europe than to those reported in less closely related avian hosts in northern Europe. All *Leucocytozoon* sequences generated in this study were deposited in NCBI and submitted to the MalAvi database (Supplementary Table 3).

Positive samples were distributed across the study region, with the northernmost at approximately 64°N (Fig. 1).

No significant relationships were found between the presence of *Leucocytozoon* infection and sex (p = 0.67), age (p = 0.08), year of collection (p = 0.076), or sampling location (p = 0.51).

### 3.2 Haemoproteus/Plasmodium spp.

The PCR for *Haemoproteus/Plasmodium* spp. resulted in positive bands for seven of 27 samples (25.9%), repeated in three independent runs (Supplementary Table 2). However, sequencing of the PCR products was unsuccessful, and infection could not be confirmed.

### 3.3 Herpesvirus and Chlamydia spp.

None of the samples tested positive for herpesvirus *or Chlamydia* spp.

## 4 Discussion

This study provides the first molecular screening of avian haemosporidian parasites in tawny owls from Norway and provides novel baseline data on *Leucocytozoon* spp. occurrence in a resident raptor species at high latitude.

This study was based on opportunistically collected carcasses, resulting in a limited number of samples overall and per category. While this restricts statistical power for detecting associations between infection status and host-related variables, it does not preclude meaningful ecological interpretation, particularly when results are evaluated in the context of existing literature.

A prevalence of 37% for *Leucocytozoon* spp. was detected, representing the first confirmed record of this genus in tawny owls in Norway. This prevalence is comparable to results reported in other European raptors: Krone et al., 2001, Krone et al., 2008a, Krone et al., 2008b documented *Leucocytozoon* prevalences ranging from approximately 20–50% in European owls; Martín-Maldonado et al. (2023) reported a prevalence of 32.1% in nocturnal raptors from central Spain; Ilgūnas et al. (2022) reported a prevalence of 43.8% in owls from Austria and Lithuania; and Santos et al. (2025) reported a higher prevalence of 63% in tawny owls from Portugal. Importantly, these studies included markedly different climatic conditions, altitudes, and hosts, yet revealed relatively comparable infection levels. Although these findings indicate that *Leucocytozoon* is not uncommon in wild birds and most infections are reported to be subclinical, it's important to have in consideration that heavy parasitaemia can cause anaemia, weakness, or death, and chronic, low-intensity infections may reduce host fitness, particularly in young animals or immunocompromised individuals (Atkinson and van Riper, 1991; Valkiūnas, 2004).

No significant associations were detected between *Leucocytozoon* infection status and sex, age class, year of collection, or sampling location. Although our sample size reduces confidence in statistical inference, the observed patterns are consistent with those reported in independent studies with larger sample sizes, supporting their biological relevance (Krone et al., 2001; Martín-Maldonado et al., 2023; Santos et al., 2025). This suggests that infection probability may be driven primarily by environmental exposure and vector availability rather than intrinsic host traits (Martínez-de la Puente et al., 2011; Palinauskas et al., 2011). This absence of strong demographic effects may also be influenced by the chronic nature of haemosporidian infections which can persist across life stages and mask demographic patterns (Ellis et al., 2014).

Environmental conditions and vector ecology play a central role in shaping haemosporidian transmission dynamics. *Leucocytozoon* spp. are transmitted primarily by black flies (Simuliidae), which depend on fast-flowing, well-oxygenated streams for larval development (Valkiūnas, 2004; Samour, 2016; Adler and McCreadie, 2019). Norway, including central regions, provides extensive suitable habitat for these vectors, which likely explains the dominance of *Leucocytozoon* in the present study. While climate change has been shown to facilitate northward expansion of *Plasmodium* and *Haemoproteus* by increasing mosquito survival and shortening sporogony time (Garamszegi, 2011; Loiseau et al., 2012; Sehgal, 2015), the implications for *Leucocytozoon* are more complex. Recent studies have demonstrated that *Leucocytozoon* infection probability and phylogenetic diversity may decrease toward lower latitudes while increasing with latitude and altitude, reflecting the ecological requirements of black fly vectors (Fecchio et al., 2018, 2020, 2023). Consequently, climate-driven changes in hydrology, precipitation, and stream dynamics may be more important determinants of *Leucocytozoon* distribution than temperature alone. The detection of *Leucocytozoon* infections in resident tawny owls at high latitude is therefore consistent with these broader patterns and underscores the need to consider vector life history when interpreting climatic effects on parasite distributions.

In contrast to *Leucocytozoon*, amplification of *Haemoproteus*/*Plasmodium* DNA could not be confirmed through sequencing and these detections were therefore not classified as true positives. However, independent PCR runs repeatedly produced clear bands of the expected size, which, together with the specificity of the nested PCR, strongly suggest the presence of low-intensity infections rather than technical artefacts. Similar difficulties in confirming haemosporidian infections by sequencing have been reported previously, particularly in samples with low parasitaemia or mixed infections (Hellgren et al., 2004; Valkiūnas et al., 2008; Krone et al., 2008a, Krone et al., 2008b). The inability to resolve these infections at the genus level highlights an important methodological limitation but does not justify disregarding the signals entirely. Mixed and coinfections with multiple haemosporidian genera are common in wildlife hosts, including raptors, and are known to complicate molecular detection due to primer competition and differential amplification success (Palinauskas et al., 2016a, 2016b; Pigeault et al., 2018; Fecchio et al., 2022). Studies employing alternative protocols and multilocus approaches have demonstrated that standard nested PCR assays may underestimate parasite diversity and fail to resolve coinfections. Future work incorporating alternative PCR protocols would be required to validate these findings and fully resolve infection complexity (Bernotienė et al., 2016; Pacheco et al., 2018).

The interpretation of these results is further supported by the ecological plausibility of their presence in the area, as both *Plasmodium* and *Haemoproteus* have been reported in other avian species in Scandinavia. While little information is known so far about the presence of avian blood parasite infections in avian species in Norway, a few case reports are available. Haemosporidians such as *Leucocytozoon*, *Haemoproteus* and *Plasmodium* have been previously detected in red-throated divers (*Gavia stellata*; Kleinschmidt et al., 2022) and in bluethroats (*Luscinia svecica*; Hellgren, 2005; Svoboda et al., 2015) in mainland northern Norway, but not detected in red-throated divers (Kleinschmidt et al., 2022), snow buntings (*Plectrophenax nivalis*; Martínez et al., 2018) or little auks (*Alle alle*; Wojczulanis-Jakubas et al., 2010) in Svalbard, a Norwegian high Arctic archipelago located above 74°N.

Overall, this study presents the first molecular screening of avian haemosporidian parasites in tawny owls from Norway and provides novel baseline data on *Leucocytozoon* spp. occurrence in a resident raptor species at high latitude. When interpreted in light of existing comparable studies, our results provide valuable baseline data from previously unsampled hosts and regions, and emphasize the need for integrative approaches combining improved molecular methods, microscopy, and expanded sampling to fully resolve haemosporidian diversity and transmission dynamics in northern raptor populations. Taken together, these findings underscore the importance of continued surveillance of avian haemosporidians and other pathogens in northern regions to detect emerging transmission patterns and evaluate potential consequences for raptor health and population dynamics.

## CRediT authorship contribution statement

**Andrea S. Ingebretsen:** Writing – review & editing, Writing – original draft, Visualization, Validation, Methodology, Investigation, Formal analysis, Data curation. **Siren C. Svanes:** Writing – review & editing, Writing – original draft, Visualization, Validation, Methodology, Investigation, Formal analysis, Data curation. **Ingvild B. Kroglund:** Writing – review & editing, Supervision, Resources, Project administration, Methodology, Funding acquisition, Conceptualization. **Oddmund Kleven:** Writing – review & editing, Methodology. **Rita Santos:** Writing – review & editing, Methodology. **Jan Eivind Østnes:** Writing – review & editing, Supervision, Funding acquisition. **Helena Costa:** Writing – review & editing, Writing – original draft, Visualization, Validation, Supervision, Project administration, Methodology, Investigation, Funding acquisition, Formal analysis, Data curation, Conceptualization.

## Funding

This study is a part of an on-going project funded as a PhD scholarship for IBK at Nord University (224000–166) and as two masters scholarships at Nord University (ASI and SCS). This work was supported by the Fundação para a Ciência e a Tecnologia grant number UI/BD/154770/2023 to RS.

## Conflict of interest

The authors declare that they have no known competing financial interests or personal relationships that could have appeared to influence the work reported in this paper.
