# Supplementary material for: Molecular evidence of haemosporidian infections in tawny owls (Strix aluco) from central Norway
Source: Int J Parasitol Parasites Wildl. 2026 Feb 9;29:101208. doi: 10.1016/j.ijppaw.2026.101208 (PMC12930077; doi:10.1016/j.ijppaw.2026.101208)
Supplement: Multimedia component 1 [file mmc1.docx]

**Supplementary information for**

**Molecular evidence of haemosporidian infections in tawny owls (*Strix aluco)* from central Norway**

Andrea S. Ingebretsen^1,*^, Siren C. Svanes^1,*^, Ingvild B. Kroglund^2^, Oddmund Kleven^3^, Rita Santos^4,5,6^, Jan Eivind Østnes^2^, Helena Costa^1,**^

^1^ Faculty of Biosciences and Aquaculture, Nord University, Universitetsalléen 11, Bodø NO–[8026](https://www.google.com/maps/place//data=!4m2!3m1!1s0x45df1760e8851fb9:0x83de2d41a44bafb9?sa=X&ved=1t:8290&ictx=111), Norway

^2^Faculty of Biosciences and Aquaculture, Nord University, Skolegata 22, Steinkjer NO–7713, Norway

^3^ Norwegian Institute for Nature Research (NINA), Høgskoleringen 9, NO-7034 Trondheim, Norway

^4^ CIBIO, Research Centre in Biodiversity and Genetic Resources, InBIO Associated Lab,Vairão Campus, University of Porto, Vairão, 4485-661, Portugal

^5^ Department of Biology, Faculty of Sciences, University of Porto, Porto, 4099-002, Portugal

^6^ BIOPOLIS Program in Genomics, Biodiversity and Land Planning, CIBIO, Vairão Campus, Vairão, 4485-661, Portugal

^*^shared authorship; ^**^corresponding author: helena.g.costa@hotmail.com

Supplementary Table 1. List of primers used in this study.

| **Target pathogen(s)** | **Primer name** | **Primer sequence (5’ to 3’)** | **Reference** |
| --- | --- | --- | --- |
| ***Haemoproteus*, *Plasmodium*, *Leucocytozoon*** (mitochondrial *cytb* gene) | HaemNFI | CATATATTAAGAGAAITATGGAG | Hellgren et al. (2004) |
|  | HaemNR3 | ATAGAAAGATAAGAAATACCATTC | Hellgren et al. (2004) |
| ***Haemoproteus-Plasmodium***  (mitochondrial *cytb* gene) | HaemF | ATGGTGCTTTCGATATATGCATG | Bensch et al. (2000) |
|  | HaemR2 | GCATTATCTGGATGTGATAATGGT | Bensch et al. (2000) |
| ***Leucocytozoon***  (mitochondrial *cytb* gene) | HaemFL | ATGGTGTTTTAGATACT TACATT | Hellgren et al. (2004) |
|  | HaemR2L | CATTATCTGGATGAGATAATG GIGC | Hellgren et al. (2004) |
| **Chlamydia**  (16S rRNA gene) | 16SG-F | TGATGAGGCATGCAAGTC | Robertson et al. (2009) |
|  | 16SG-R | TTACCTGGTACGCTCAAAT |  |
| **Herpesvirus** (DNA polymerase gene) | DFA | GAYTTYGCNAGYYTNTAYCC | VanDevanter et al. (1996) |
|  | ILK | TCCTGGACAAGCAGCARNYSGCNMTNAA |  |
|  | KG1 | GTCTTGCTCACCAGNTCNACNCCYTT |  |
|  | TGV | TGTAACTCGGTGTAYGGNTTYACNGGNGT |  |
|  | IYG | CACAGAGTCCGTRTCNCCRADAT |  |

Supplementary Table 2. Details of each individual, including sex, age class, location and date of delivery, cause of death and PCR and sequencing results.

| **ID** | **Sex** | **Age class** | **Location** | **Date** | **Cause of death** | **PCR positive to**  **(sequencing results)** |
| --- | --- | --- | --- | --- | --- | --- |
| **ID1** | Female | Adult | Levanger | 5/10/2017 | - | None |
| **ID2** | Female | Adult | Steinkjer | 7/02/2017 | Hit by car | *Leucocytozoon* (STAL3) |
| **ID3** | Female | 2C | Orkdal | 11/02/2018 | - | None |
| **ID4** | Female | Adult | Steinkjer | 21/09/2017 | - | None |
| **ID5** | Male | Adult | Rennebu | 30/03/2009 | - | *Leucocytozoon* (STAL1); *Haemoproteus-Plasmodium*  (failed sequencing) |
| **ID6** | Male | Adult | Vormstad | Unknown | - | None |
| **ID7** | Female | Adult | Verdal | 5/12/2016 | - | None |
| **ID8** | Female | Adult | Orkdal | 15/02/2009 | Predation | *Leucocytozoon* (STAL3) |
| **ID9** | Male | 2C | Selbu | 26/01/2016 | - | None |
| **ID10** | Female | Adult | Trondheim | 18/03/2018 | - | None |
| **ID11** | Female | Adult | Levanger | 29/09/2019 | - | *Leucocytozoon* (STAL1); *Haemoproteus-Plasmodium*  (failed sequencing) |
| **ID12** | Female | Adult | Vormstad | - | - | *Leucocytozoon* (STAL3); *Haemoproteus-Plasmodium*  (failed sequencing) |
| **ID13** | Female | 1C | Trondheim | 15/05/2017 | - | None |
| **ID14** | Male | Adult | Verdal | 18/09/2011 | - | None |
| **ID15** | Female | Adult | Selbu | 13/07/2017 | - | None |
| **ID16** | Male | 1C | Trondheim | 05/05/2017 | - | None |
| **ID17** | Female | Adult | Inderøy | 04/09/2013 | - | None |
| **ID18** | Female | Adult | Levanger | 05/05/2020 | Electrocution | *Leucocytozoon* (STAL3); *Haemoproteus-Plasmodium*  (failed sequencing) |
| **ID19** | Male | Adult | Verran | 25/10/2019 | Hit by car | *Leucocytozoon* (STAL1); *Haemoproteus-Plasmodium*  (failed sequencing) |
| **ID20** | Male | 1C | Levanger | 31/05/2020 | - | None |
| **ID21** | Male | 1C | Inderøy | 12/05/2020 | Predation | None |
| **ID22** | Female | Adult | Verdal | 01/02/2020 | Trap | None |
| **ID23** | Female | Adult | Levanger | 13/10/2021 | Hit by car | *Leucocytozoon* (STAL3); *Haemoproteus-Plasmodium*  (failed sequencing) |
| **ID24** | Female | Adult | Verdal | 12/05/2021 | - | *Leucocytozoon* (STAL3); *Haemoproteus-Plasmodium*  (failed sequencing) |
| **ID25** | Female | Adult | Stod | 08/05/2022 | - | *Leucocytozoon* (STAL3) |
| **ID26** | Female | - | - | 01/05/2022 | - | None |
| **ID27** | Female | Adult | Rinnleiret | 19/09/2022 | Trap | None |

*- : Unknown or data not available; 1C: first calendar year; 2C: second calendar year*

Supplementary Table 3. List of NCBI accession numbers from the sequences obtained in this study.

| **Sample ID** | **Accession number** |
| --- | --- |
| **ID2** | PX663933 |
| **ID5** | PX663934 |
| **ID8** | PX663935 |
| **ID11** | PX663936 |
| **ID12** | PX663937 |
| **ID18** | PX663938 |
| **ID19** | PX663939 |
| **ID23** | PX663940 |
| **ID24** | PX663941 |
| **ID25** | PX663942 |
